# BUILDING CAPACITY FOR PATIENT-CENTERED OUTCOMES RESEARCH IN THE AGING RESEARCH COMMUNITY

**DOI:** 10.1093/geroni/igac059.1832

**Published:** 2022-12-20

**Authors:** Taylor Gray, Sophia Webber, Erin McGaffigan

**Affiliations:** UMass Boston, Waltham, Massachusetts, United States; University of Wisconsin- Madison, Madison, Wisconsin, United States; UMASS Boston Gerontology Institute, North Reading, Massachusetts, United States

## Abstract

Patient-centered outcomes research (PCOR) models are increasingly used to ensure stakeholders inform evidence-based systems of care. Unfortunately, older adults, especially those of low income, frailty, and color, are often left out of PCOR, leading to continued health and service disparities. The Aging PCOR Learning Collaborative was funded by the Patient-Centered Outcomes Research Institute in 2020 to conduct a series of multi-media education, training, and mentoring activities to change how older adults are viewed and engaged in research. This project was strongly guided by multiple advisory structures, including two stakeholder advisory boards, two older adult paid advisors, and two student advisors, all of whom assumed an integral role in the design and implementation of project activities, dissemination of products, and analysis and reporting of evaluation methods. Project staff applied a multi-domain evaluation framework to analyze data relevant to these activities, which demonstrated the effective role of advisory structures and the project’s reach to over 300 older adults, researchers, funders, and academic leaders. This session will review the steps required to implement these activities as well as our project reach, outputs, and mid-term outcomes, providing one of the first glimpses into how to measure this significant shift in research paradigm.

